# Emotional Tone, Analytical Thinking, and Somatosensory Processes of a Sample of Italian Tweets During the First Phases of the COVID-19 Pandemic: Observational Study

**DOI:** 10.2196/29820

**Published:** 2021-10-27

**Authors:** Dario Monzani, Laura Vergani, Silvia Francesca Maria Pizzoli, Giulia Marton, Gabriella Pravettoni

**Affiliations:** 1 Department of Oncology and Hemato-Oncology University of Milan Milan Italy; 2 Applied Research Division for Cognitive and Psychological Science IEO, European Institute of Oncology IRCCS Milan Italy

**Keywords:** internet, mHealth, infodemiology, infoveillance, pandemic, public health, COVID-19, Twitter, psycholinguistic analysis, trauma

## Abstract

**Background:**

The COVID-19 pandemic is a traumatic individual and collective chronic experience, with tremendous consequences on mental and psychological health that can also be reflected in people’s use of words. Psycholinguistic analysis of tweets from Twitter allows obtaining information about people’s emotional expression, analytical thinking, and somatosensory processes, which are particularly important in traumatic events contexts.

**Objective:**

We aimed to analyze the influence of official Italian COVID-19 daily data (new cases, deaths, and hospital discharges) and the phase of managing the pandemic on how people expressed emotions and their analytical thinking and somatosensory processes in Italian tweets written during the first phases of the COVID-19 pandemic in Italy.

**Methods:**

We retrieved 1,697,490 Italian COVID-19–related tweets written from February 24, 2020 to June 14, 2020 and analyzed them using LIWC2015 to calculate 3 summary psycholinguistic variables: emotional tone, analytical thinking, and somatosensory processes. Official daily data about new COVID-19 cases, deaths, and hospital discharges were retrieved from the Italian Prime Minister's Office and Civil Protection Department GitHub page. We considered 3 phases of managing the COVID-19 pandemic in Italy. We performed 3 general models, 1 for each summary variable as the dependent variable and with daily data and phase of managing the pandemic as independent variables.

**Results:**

General linear models to assess differences in daily scores of emotional tone, analytical thinking, and somatosensory processes were significant (F_6,104_=21.53, *P*<.001, R^2^= .55; F_5,105_=9.20, *P*<.001, R^2^= .30; F_6,104_=6.15, *P*<.001, R^2^=.26, respectively).

**Conclusions:**

The COVID-19 pandemic affects how people express emotions, analytical thinking, and somatosensory processes in tweets. Our study contributes to the investigation of pandemic psychological consequences through psycholinguistic analysis of social media textual data.

## Introduction

### Background

As a way to express information, news, opinions, and even private emotions and to connect people worldwide, Twitter, established in 2006, is a microblogging service that is the 13th most-used social media platform, with 340 million users [1]. In the first quarter of 2020, it registered 166 million average monetizable daily active users, with a 24% growth from 2019 [2]. And Twitter itself attributes part of this exceptional growth to a “global conversation related to the COVID-19 pandemic” [3]. While the coronavirus disease pandemic is affecting the world, regional and national lockdowns are restraining the possibility to travel and physically meet other people: Social networks, including Twitter, now represent a way to keep in touch, exchange information, solve problems, and conversate together and globally. And there is even something more.

Infodemiology is a new research field using online data and defined as “the science of distribution and determinants of information in an electronic medium, specifically the internet, or in a population, with the ultimate aim to inform public health and public policy” [4]. Among the infodemiology indicators, “metrics on the ‘chatter’ in discussion groups, blogs, and microblogs (eg, Twitter)” [4] are considered. Along this line, various researchers have successfully used this kind of data in the health context. Specifically, Twitter represents a unique opportunity for scholars to recruit participants, deliver interventions, or directly obtain data [5]. In particular, as a data source, it can provide population-level, real-time, high-volume, easily, publicly accessible data [5]: These are beneficial features, especially in the psychosocial field that normally relies on self-report, time-lagged questionnaires, with limited numbers of participants.

Today, Twitter-based health research represents a rapidly developing field, combining different methodologies and applying to various contexts, such as public health, infectious diseases including Ebola and influenza, neurology, and psychiatry [5]. Some studies have also been conducted in the COVID-19 pandemic context, demonstrating the feasibility of using Twitter as a means to collect valuable data to obtain deep insights in this emergency situation. Lwin and colleagues [6] collected more than 20 million tweets written worldwide during the first phases of the pandemic and studied the emotional responses to COVID-19 by using sentiment analysis. Xue and colleagues [7] used sentiment analysis alongside unsupervised machine learning and qualitative methods to identify main COVID-19–related themes discussed on Twitter, such as news, cases, and deaths, accompanied by a sentiment of fear.

Other studies, instead, relied on psycholinguistic analysis of Twitter data. Su and colleagues [8] used psycholinguistic analysis on Weibo and Twitter posts to investigate the psychological impact of lockdown measures in China and Italy: After lockdown, people used more cognitive processes and home words.

Indeed, as demonstrated by a vast amount of literature [9], the words we use in our daily lives have various links to different psychosocial variables, including mental health, psychological status, and “ongoing emotional and cognitive coping processes, and idiosyncratic reactions to crisis” [10]. In fact, the pandemic could be considered as “[…] the cause of individual and collective traumas” [11], that is, also having tremendous consequences on mental and psychological health [12,13].

Overall, psycholinguistic analysis of textual data coming from Twitter allows some advantages. Usually, assessing psychological variables requires the recruitment of a sample of participants, relying on their availability to individually administer questionnaires and instruments. This process is expensive and time-consuming, resulting in a limited amount of data, often biased by the issues associated with self-report instruments, such as a time lag between the event of interest experienced by people and the moment of data collection. Psycholinguistic analysis of Twitter data requires downloading, in a quite fast and automatic way, a massive amount of population-level data in near real time—as tweets written immediately after the event of interest—in a discreet and unobtrusive way, resulting in a faster and less expensive process.

Among the psychological variables, psycholinguistic analysis of textual Twitter data could provide information about emotional expressions, analytical thinking, and somatosensory processes, which are particularly important in traumatic event contexts.

Specifically, emotional tone is a psycholinguistic variable that summarizes the presence of positive and negative emotions in written text as the difference between positive-emotion words and negative-emotion words [10]. Individuals’ expressions of emotions in language are connected to the way they experience the world and also react to, and cope with, traumatic events [9]. In particular, experiencing positive emotions after a challenging event is important for resilience [14], while some studies highlighted how, after a traumatic experience such as the September 11 attacks, the emotional tone in journal entries by people in the United States was low, in other words characterized by a negative tone, which slowly rebuilt after some time [10].

Analytical thinking is a psycholinguistic variable that reflects “the degree to which people use words that suggest formal, logical, and hierarchical thinking patterns” [15]. A lower level of analytical thinking reflects a more narrative and personal thinking pattern. The value of cognitive words in trauma narratives remains controversial: These types of words are linked with positive or negative effects on people’s well-being [16].

In trauma narratives, somatosensory words, such as words related to body, sensory, and perceptual processes, assume great relevance, with a stronger presence than in other neutral or positive-tone narratives [17]. The use of this type of words is associated with the symptoms of posttraumatic stress disorder (PTSD) and depression [17-19].

Our aim was to analyze the influence of the pandemic—such as official Italian COVID-19 daily data (new cases, deaths, and hospital discharges) and the phase of managing the pandemic—on psycholinguistic variables in Italian tweets written during the first phases of the COVID-19 pandemic in Italy.

### Objective

The pandemic is characterized by daily information about new cases and deaths and by governments’ decisions and restrictions that impact everyone’s lives: It could be considered a collective and individual traumatic experience [11]. This traumatic experience can have profound psychological consequences on mental health and the well-being of citizens that can also be reflected, as discussed earlier, in people’s use of words, specifically the emotional tone, analytical thinking, and somatosensory processes variables.

Our aim was to analyze the way people express emotions, their analytical thinking, and somatosensory processes in a sample of Italian tweets during the first phases of the COVID-19 pandemic in Italy. Specifically, we were interested in assessing the influence of official Italian COVID-19 daily data (eg, new cases, new deaths, and hospital discharges) as well as the phase of managing the outbreak on tweets occurring during the following 24 hours, specifically on the emotional tone, analytical thinking, and somatosensory processes in tweets.

## Methods

### Dataset

The dataset used in this study came from a large-scale COVID-19 Twitter chatter project that actively collected COVID-19 tweets from January 1, 2020 (for a brief overview, see [20]). Specifically, this dataset, which has been made freely available by Banda and colleagues [20] through Zenodo, includes tweets collected from the publicly available Twitter Stream API with a collection process that gathered any available tweets with keywords related to COVID-19 (eg, “coronavirus,” “2019ncov,” “COVID19,” “COVID-19”). See [20] for further information on the full list of keywords and the rationale for their selection and inclusion. As of September 20, 2020, this project had collected almost 166 million unique tweets. The project only released the Tweet IDs of the collected tweets; thus, the software DocNow Hydrator was used to extract tweets. This user-friendly software has been proven effective by previous research [21,22]. We only selected tweets in the Italian language created between 6:00 pm on February 24, 2020 and 11:59 pm on June 14, 2020. Both the language and timestamp of tweets are provided directly by Twitter through its API, a tool to contribute to, engage with, and analyze the conversation happening on Twitter. We chose to focus on this period because official data about the COVID-19 outbreak were available since 6:00 pm on February 24, 2020 (ie, 3 days after Italian Patient One was tested positive), and “Phase 3” started on June 15, 2020, characterized by a sharp loosening of previous public health measures and restrictions.

In addition, official data about daily new cases, new deaths, and new discharges from hospital were also retrieved from the GitHub page of the Italian Prime Minister's Office and Civil Protection Department. From February 24, 2020 to April 17, 2020, data on the COVID-19 outbreak in Italy were communicated in a press conference held daily at 6:00 pm by the head of the Civil Protection Department. After April 17, 2020, the daily press conference was no longer held, but official information about the pandemic continued to be released at 6:00 pm through a daily bulletin.

We considered 3 different phases of managing COVID-19, characterized by distinct restrictions and measures to counteract virus spreading. The first was the outbreak, from February 24, 2020 (ie, the day on which the official Civil Protection Department 6:00 pm press conference began) to March 8, 2020: Along with the first confirmed indigenous cases, regional and national governments began to take action, including school and university closures, postponing or canceling some public events, and strict lockdown for 11 municipalities in northern Italy. The second was Phase 1, from March 9, 2020 to May 3, 2020: A “I stay home” national decree imposed lockdown in all Italian regions, and citizens were allowed to leave their homes only for documented work, health, or emergency reasons, while nonessential commercial activities were closed. The third was Phase 2, from May 4, 2020 to June 14, 2020: A gradual relaxing of lockdown restrictions began, with reopening of some services and activities, such as parks, museums, restaurants, and bars for take-away service; practicing social distance remained mandatory.

Data use complied with ethical guidelines for internet research [23]. The European Union General Data Protection Regulation 2016/679 allows for the use of anonymous data for research purposes under certain conditions. Since all analyses have been performed on public and anonymized meta-data, no institutional review board approval was required for the use of this database or the completion of this study.

### Statistical Analysis

Text mining and text analysis were performed with R version 3.4.3 and Linguistic Inquiry and Word Count (LIWC) 2015. We were interested in understanding whether daily data on the COVID-19 outbreak would affect how people express emotion, cognition, and somatosensory processes in their tweets during the following 24 hours. Thus, before analysis, all tweets were preprocessed: Daily tweets from 6:00 pm to 5:59 pm the following day were merged into a single text file. For instance, the overall corpus for March 1, 2020 included aggregated text coming from 11,707 tweets from 6:00 pm on March 1 to 5:59 pm on March 2. There was a total of 1,692,181 tweets from 6:00 pm on February 24, 2020 to 23:59 pm on June 14, 2020; the number of tweets per day ranged from 6977 (on June 14, 2020) to 33,356 (on May 25, 2020) with a daily average of 15,108.76 (SD 3895.29) tweets.

Then, each daily text was analyzed with the Italian LIWC2007 Dictionary [24] and the Italian Function Words Dictionary 2015 of LIWC2015 [25]. LIWC calculates the percentage of total words in each text that falls into predefined linguistic and psycholinguistic categories. We then computed separate indexes for emotional tone, analytical thinking, and somatosensory processes. Based on previous research, each of these 3 summary variables are constructed from different LIWC categories. First, to calculate the emotional tone score, we employed the procedure described by Cohn et al [10]. Specifically, tone was computed as (positive emotion) – (negative emotion): thus, the higher the score, the more positive the emotional tone of daily tweets. Second, analytical thinking is a factor-analytically derived dimension based on 8 function word dimensions. This dimension “captures the degree to which people use words that suggest formal, logical, and hierarchical thinking patterns” [15]. It was computed as (articles) + (prepositions) - (total pronouns) - (auxiliary) - (negations) - (conjunctions) - (adverbs) [26]: the higher the score, the higher the analytical thinking of the daily tweets. Third, as somatosensory details, in particular words related to body and perception, have been found to be common and important in different studies examining trauma narratives [16], we decided to calculate a somatosensory index, namely somatosensory processes: This index was computed as (perceptual processes) + (body). These 2 categories captured the use of words related to perceptual experiences (such as “observing, heard, feeling, rumors, touch”) and body parts, processes, or diseases (such as “cheek, hands, spit, cough, flesh, brain, hearth, pain, contagious, headache, sick”), tapping into perceptual and sensory features that are meant to be common in this type of narrative. Higher scores in this index imply higher somatosensory experiences expressed in daily tweets. Since emotional tone, analytical thinking, and somatosensory processes were computed for each day by considering all the text coming from daily tweets, in all subsequent analyses, the total sample was the number (ie, 122) of days from February 24, 2020 to June 14, 2020 (with days as the unit of analysis).

We performed 3 general linear models using Jamovi 1.1 [27,28], 1 for each of the 3 LIWC summary variables, namely emotional tone, analytical thinking, and somatosensory processes. In each model, the LIWC summary variable was entered as the dependent variable; daily official data about new cases of COVID-19, new deaths, and new discharges were entered as continuous independent variables, while the phase of managing the COVID-19 outbreak was entered as a categorical independent variable (coded as 1=COVID-19 spreading; 2=Phase 1; 3=Phase 2). Specifically, these general linear models assessed whether daily new cases, new deaths, and new hospital discharges, alongside the phases of managing the COVID-19 pandemic, influenced the 3 daily summary variables constructed through LIWC. Besides the main effects, we included second- and third-order interaction terms for the continuous independent variable. We adopted a stepwise backward regression analysis approach. Thus, starting from the full model, nonsignificant, higher-order terms were eliminated one at a time, in order to obtain a final, more parsimonious model. If not one of the interaction terms was significant, the final model included only the main effects of all the predictors. For significant interactions, simple slope analysis was performed to test the effect of a specific predictor at different levels (ie, 1 standard deviation above and below the mean) of another predictor. All continuous independent variables were mean centered. The magnitude of each effect was interpreted by considering its associated partial eta squared (ie, η_p_^2^). Specifically, effects were considered weak (.01 < η_p_^2^ ≤ .06), moderate (.06 < η_p_^2^ ≤.14), or strong (η_p_^2^ > .14). The final dataset and the scripts to perform data analysis are available in Multimedia Appendix 1 and Multimedia Appendix 2, respectively.

## Results

**Figure 1:**
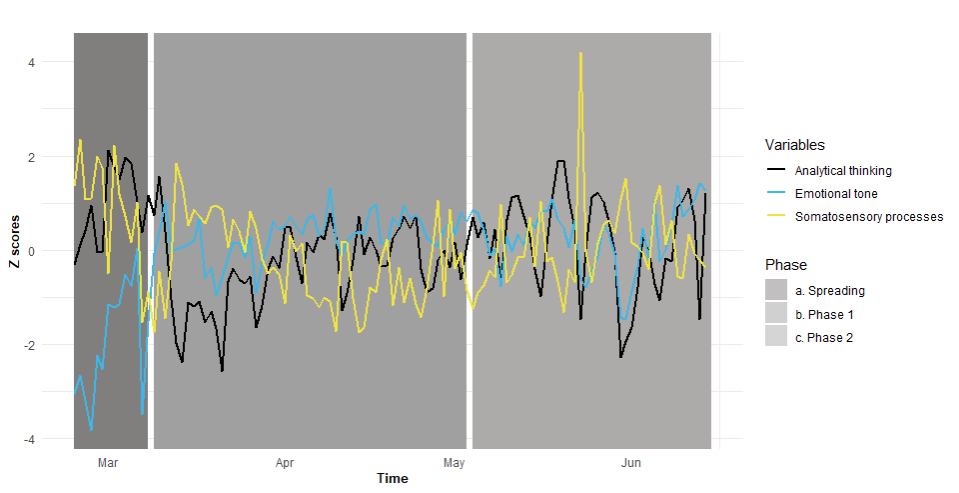
Trends over time for emotional tone, analytical thinking, and somatosensory processes.

Figure 1 displays the trends over time for emotional tone, analytical thinking, and somatosensory processes (as z scores) as expressed in daily tweets from February 24, 2020 to June 14, 2020.

**Figure 2:**
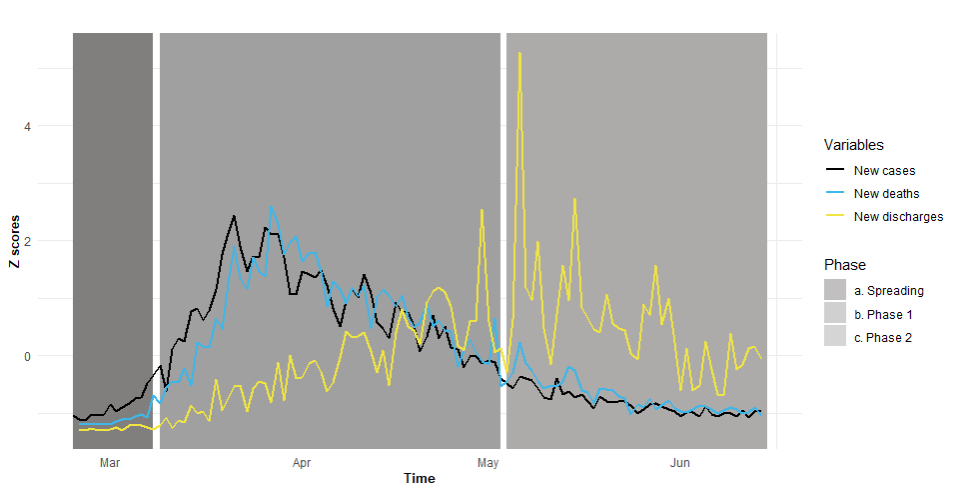
Trends over time for daily new cases, new deaths, and new hospital discharges.

Figure 2 displays trends over time for daily new cases, new deaths, and new hospital discharges (as z scores) from February 24, 2020 to June 14, 2020.

Results of the 3 general linear models assessing influences on each of the 3 LIWC summary variables, namely emotional tone, analytical thinking, and somatosensory processes, are reported in Table 1. By considering emotional tone, the final general linear model was significant (F_6,104_=21.53, *P*<.001) and explained more than 55% of the dependent variable. Specifically, we found a significant interaction between daily new cases and new deaths for COVID-19 in explaining emotional tone (F_1,104_=4.10, β=–.24, *P*=.045, η_p_^2^=.04). The simple slope analysis showed that, when the number of deaths was low (b=–0.00, SE=0.00, t_104_=–0.13, *P*=.900) or average (b=–0.00, SE=0.00, t_104_=–1.27, *P*=.207), daily new cases of COVID-19 were not related to tone. On the other hand, when the number of deaths was high, the higher the number of daily new cases, the lower the estimated emotional tone was (b=–0.00, SE=0.00, t_104_=–2.42, *P*=.017). Other interactions were not significant and, thus, were excluded one at a time by adopting a stepwise backward regression analysis approach. The main effect of daily new cases was not significant (F_1,104_=1.62, β=–.27, *P*=.207, η_p_^2^=.02), while the effect of daily new deaths was significant but weak (F_1,104_=3.63, β=.41, *P*=.048, η_p_^2^=.03). Moreover, emotional tone was not related with daily number of new hospital discharges (F_1,104_=0.12, β=–.03, *P*=.729, η_p_^2^=.00). Finally, phases of managing the COVID-19 outbreak were responsible for strong differences in emotional tone (F_2,104_=30.27, *P*<.001, η_p_^2^=.37). Estimated marginal means of daily scores of emotional tone were –1.08 (SE=0.04) during the outbreak, –0.83 (SE=0.02) during Phase 1, and –0.83 (SE=0.03) during Phase 2. As highlighted by post hoc analyses with a Bonferroni correction (*P*<.05), daily scores of emotional tone during the first outbreak were lower than the ones reported in both Phase 1 and Phase 2. The 2 latter phases did not differ in daily scores of tone.

**Table 1:** Summary of the results of the 3 general linear models.

Variables	Emotional tone^a^				Analytical thinking^b^				Somatosensory processes^c^		
	β	*P* value	η_p_^2^	β		*P* value	η_p_^2^	β		*P* value	η_p_^2^
Phase	-^d^	<.001	0.37	-^d^		.001	0.12	-^d^		.55	0.01
New cases	–.27	.21	0.02	–.84		.001	0.10	.51		.07	0.03
New deaths	.41	.06	0.03	.55		.02	0.05	–1.01		<.001	0.12
New discharges	–.03	.73	0.00	.10		.33	0.01	.11		.38	0.01
New case*New deaths	–.24	.045	0.04	-^d^		-^d^	-^d^	.44		.004	0.08

^a^F_6,104_=21.53, *P*<.001, R^2^=.55.

^b^F_5,105_=9.20, *P*<.001, R^2^=.30.

^c^F_6,104_=6.15, *P*<.001, R^2^=.26.

^d^Not applicable.

The final general linear model performed to assess differences in daily scores of analytical thinking was significant (F_5,105_=9.20, *P*<.001) and explained 30% of the dependent variable. No significant second- and third-order interactions were observed; thus, all interaction terms were excluded one at a time by adopting a stepwise backward regression analysis approach. Analytical thinking was not related to daily new discharges from hospital (F_1,105_= 0.95, β=.10, *P*=.332, η_p_^2^=.01), while it was negatively and moderately related to daily new cases of COVID-19 (F_1,105_=11.14, β=–.84, *P*=.001, η_p_^2^=.10) and positively but weakly linked to new deaths related to COVID-19 (F_1,105_=5.48, β=.55, *P*=.021, η_p_^2^=.05). Daily scores of analytical thinking differed moderately among different phases of managing the COVID-19 outbreak (F_1,105_=7.27, *P*=.001, η_p_^2^=.12). Estimated marginal means for daily scores of analytical thinking were –2.14 (SE=0.51) during the outbreak, –4.01 (SE=0.29) during Phase 1, and –4.22 (SE=0.32) during Phase 2. As highlighted by post hoc analyses with a Bonferroni correction (*P*<.05), daily scores of analytical thinking during the first outbreak were lower than the scores reported in both Phase 1 and Phase 2. The 2 latter phases did not differ in daily scores of analytical thinking.

By considering somatosensory processes, the final general linear model was significant (F_6,104_=6.15, *P*<.001) and explained more than 26% of the dependent variable. Specifically, we found a significant interaction between daily new cases and new deaths related to COVID-19 in explaining somatosensory processes (F_1,104_=8.79, β=.44, *P*=.004, η_p_^2^=.08). The simple slope analysis showed that, when the number of deaths was low (b=0.00, SE=0.00, t_104_=0.19, *P*=.851) or average (b=0.00, SE=0.00, t_104_=1.87, *P*=.065), daily new cases of COVID-19 were not related to somatosensory processes. On the other hand, when the number of deaths was high, the higher the number of daily new cases, the higher the estimated score of somatosensory processes was (b=0.00, SE=0.00, t_104_=3.55, *P*<.001). Other interactions were not significant and, thus, were excluded one at a time by adopting a stepwise backward regression analysis approach. The main effect of daily new cases was not significant (F_1,104_=3.48, β=.51, *P*=.065, η_p_^2^=.03), while the main effect of daily new deaths was significant and moderate (F_1,104_=13.69, β=–1.01, *P*<.001, η_p_^2^=.12). Moreover, daily number of new hospital discharges was not related with somatosensory processes (F_1,104_=0.77, β=.11, *P*=.383, η_p_^2^=.01). Finally, phases of managing the COVID-19 outbreak were not responsible for differences in somatosensory processes (F_2,104_=0.60, *P*=.551, η_p_^2^=.01).

## Discussion

### General Considerations

All 3 general linear models to assess differences in daily scores of analytical thinking, emotional tone, and somatosensory processes were significant, with specific and different patterns.

As already pointed out, we might discuss our results considering this pandemic as “the cause of individual and collective traumas” [11]. In fact, different people dealing with the same stressful event could develop various reactions: Some individuals could develop a nonpathological response, with emotional, cognitive, and physical symptoms resolving spontaneously after some days or weeks, the successful implementation of resilience and coping strategies, and a return to a previous baseline without long-lasting consequences. For these individuals, the stressful event remains only “potentially” traumatic. Other individuals, instead, develop more pathological reactions, ranging from adjustment disorders to PTSD, with trauma lived as “a complex emotional response to a stressful event, that overwhelms the individual’s capacity to cope” [11].

Various studies have analyzed individuals’ language use after a traumatic event (eg, Cohn et al [10]), but, to the best of our knowledge, this is the first study using these summary variables in a sample of Italian tweets during the first phases of the pandemic. First, in all our general models, we did not find any significant effect of the daily number of new hospital discharges on our variables of interest. Daily hospital discharges, compared to daily new cases and new deaths, was the only “positive” data considered. The absence of any effect could be due to the negativity bias, which is the human tendency to give more importance and attention to negative data—or entities in general—[29] such as COVID-19 deaths and new cases, while ignoring positive data, such as hospital discharges. The negativity bias has been demonstrated to be related to life stressors and PTSD [30,31], as individuals affected by PTSD tend to focus their attention on potential threats [32]. This could also explain the fact that, in each model, summary variables were related to the negative data. So, when experiencing a stressful event, such as the pandemic period, individuals may experience negativity bias, focusing more on negative data. Experiencing these data could be considered a stressful event. In particular, we found that increases in daily deaths and daily new cases, in other words the worst situation possible, increased negative emotional tone. This seems intuitive: Negative emotion words are habitually used when writing about a negative event, such as the situation described before, and have been linked with suicide and depression [9]. Moreover, negative alterations in mood experience, negative affect, and difficulty in experiencing positive emotions are typical reactions experienced after a stressful event and, in some cases, could be symptoms of PTSD [33].

The same interaction was found to have an effect on increased use of somatosensory words. This result also seems intuitive, as the use of sensory, body, and perceptual words in narratives related to traumatic events are common and often linked to PTSD and its symptoms, even in studies of more individual traumatic events such as traffic accidents [16,18].

Regarding analytical thinking, we found 2 opposite effects: Daily new cases were negatively linked with this linguistic marker, while new deaths were positively linked with it. High scores in the analytical thinking variable are related to a formal and logical thinking pattern, while a low score is related with a more narrative style, focused on the here and now [15]. Various studies have considered the use of cognitive words after traumatic events: More cognitive words are often present in trauma- or distress-related narratives [34]. Using cognitive words is linked to an individual’s effort to elaborate and integrate the event in their own memories [16], reflecting “an active search for meaning and understanding of the stressful event” [34]. In fact, using cognitive words, in particular causal and insight ones, when writing about a past event is linked to “the active process of reappraisal” [9]. So, using more cognitive words is associated with better physical health [35,36], fewer PTSD symptoms [37], and adaptive coping strategies [38]. On the contrary, some studies have shown a link between cognitive words and PTSD symptoms [39]. In fact, as some authors pointed out [39,40] using LIWC, it is difficult to understand how these words are used, for example referring to “organized or disorganized thoughts” or linked to “ruminative processes and fruitless attempts to assimilate what happened” [16]. After a traumatic event, indeed, individuals’ thoughts could be affected in different ways. For instance, PTSD symptoms include intrusive and upsetting memories or negative thoughts about themselves and the world or avoidance of thoughts related to trauma [33]. These different reactions and discordance about the meaning of cognitive words after traumatic events may account for these opposite effects. Even if more data and research are needed, we may cautiously think that, when confronted with new deaths data—the worst news—individuals may try to react using a formal and logical way of writing, trying to make sense of this negative information. Considering new cases data, so slightly less negative, people may try to avoid the data or react with a more narrative tone, feeling less the need to elaborate them.

The last interesting result we retrieved is the effect of the phase of managing the pandemic on emotional tone and analytical thinking variables. In particular, both emotional tone and analytical thinking were lower during the outbreak, then increased in the first and second phases. As explained, the initial phase of the pandemic in Italy was characterized by different restrictions and measures taken by the government in order to counteract the spread of the virus. These measures differed, in particular, between the outbreak and the first and second phases. As the first indigenous cases were confirmed at the end of February, but maybe the gravity of the situation was still not clear, different day-to-day actions and initiatives were taken in each part of Italy: Universities and schools were closed first only in northern regions and initially only for some days; 11 municipalities in Lombardy and Veneto were in strict lockdown; some major public events, such as the Carnival of Venice, were postponed or cancelled; in other regions, considered at minor risk, schools remained open with some events confirmed, such as Series A soccer matches with the presence of fans in southern Italy. However, contradictory messages hit the population: Fake news stating the closure of all Italian schools circulated at the end of February, while some ads and initiatives reassured people, even in the northern areas, to continue to live their normal lives; all of this contributed to creating a climate of uncertainty. The first and second phases, instead, were characterized by national-level and long-term measures, with a strict lockdown and suspension of nonnecessary activities in all Italian regions, which gradually loosened at the beginning of May. These 2 phases marked a tragic and dramatic situation but were more stable and predictable in their restrictions. These differences between the very first and the other phases could account for the differences retrieved in our summary variables. Uncertainty about future events, as people may experience during the outbreak phase about future restrictions and development of the emergency, is common in threat contexts and could elicit negative emotions, such as anxiety and fear [41]. After the situation became more stable in the subsequent phases, with less uncertainty, emotional tone may increase. This emotional tone pattern confirms other results retrieved in the COVID-19 pandemic and in other trauma contexts: Sadiković and colleagues [42] found decreased worry, fear, and boredom over 5 weeks after the first COVID-19–confirmed case in Serbia. Cohn and colleagues [10] found that, immediately after the September 11 attacks, emotional tone measured in a sample of online journals was low, returning slowly to baseline after 1 week. Experiencing negative emotions is a typical reaction after an emotional upheaval and uncertain and threatening situation, even representing a specific criterion for PTSD disorder [33]; experiencing positive emotions after a crisis acts as a buffer against depression in resilient individuals [14], and positive emotions in trauma narratives are linked to better adaptation or less severe PTSD symptoms [37,43]. So, after the initial, negative reaction, the situation changed, becoming more predictable and less uncertain, and people enact their resilience and coping strategies, using more positive emotions to overcome the emotional upheaval and resulting again in a more positive way of expressing themselves.

The uncertainty of the outbreak situation—with different restrictions and even contradictory circulating messages—may also have had an impact on people's analytical thinking and use of words: Reasoning and trying to make sense of events are difficult in such contexts [41]. People might have reacted with a more logical thinking style, trying to find meaning from the situation only during the first and second phases when things were more stable, the gravity and seriousness of the emergency became clearer, and a consistent view was reached. This result seems in contrast with the one obtained by Cohn and colleagues [10], who highlighted a rapid increase in cognitive word use immediately after the attacks; their level returned to baseline after some days and then decreased again. We have to point out that our study and the study by Cohn et al [10] used different writing samples (tweets vs journal entries) and also different words in the analysis: Even if theoretically tapping the same construct, such as a sort of thinking style, analytical thinking is based on function words while the cognitive processing index used by Cohn and colleagues [10] reflects words such as because, think, and question. However, we think that these differences in results could be due to the reasons already explained: September 11 was a punctual, intense, and disruptive outbreak, leading to a rapid need to make sense of what was happening. This pandemic outbreak phase, instead, was very different, with a slower unravelling and uncertainty that persisted for weeks and weeks.

As COVID-19 could be considered “the cause of individual and collective traumas” [11], we discussed our results considering previous studies both concerning individual (for example, traffic accidents or relationship breakups [18,34]) and collective trauma (for example, the September 11 attacks [10]). With heterogeneous yet similar consequences for individuals, more research is needed to highlight pandemic-specific psycholinguistic trauma at both individual and collective levels.

### Limitations

Our study is not exempt from certain limitations. Our data consist of publicly available Italian tweets, so our results could not be generalized to other Twitter users with private accounts nor to the general Italian population. Even if it is used by a considerable amount of people—3.7 million users as of January 2020—Twitter is now only the sixth most used social media platform in Italy.

Moreover, we did not collect any information about users actually writing the analyzed tweets: Some demographic and other characteristics (eg, gender, age, working status, coping strategies) could account for differences in reactions to official COVID-19 data and for different use of words in their tweets. Specifically, some studies showed that even the area from which people tweet could account for some differences in their tweets: Gore et al [44], for example, showed that geotagged tweets in US areas with lower obesity rates have, among other results, a higher level of happiness. Another study [45] found that weather, days, and type of activities done during the day impact on emotions expressed in tourists’ tweets.

So, specifically regarding our context, we might think that urban areas and their characteristics, days, and seasonal weather could have influenced the emotional tone and, globally, the words people use in their tweets.

### Implications and Future Work

We think that our study could have relevant implications for actionable policies in the health care context and for future related works expanding our research questions.

These results prove the feasibility and importance of infodemiological indicators and psycholinguistic analysis to monitor mental health–related variables in a fast and cost-effective way. While traditional psychology instruments and measures (such as self-reported questionnaires and surveys) provide a one-time measure of the variable of interest in a limited sample, this method could provide longitudinal and population-level data. Considering all the limitations and influences, this method could be used as active surveillance of the impact of a pandemic and the related daily sharing of information on people’s mental health, providing dynamic knowledge to inform relevant health policies. Knowing in advance or in real time which type of information—as new daily cases, new daily deaths, or the phase of the pandemic—could have an impact and how it impacts emotions, analytical thinking, and the mental health of a population could allow the implementation of ad hoc and concrete responses. As a pandemic is constantly and heavily affecting our daily lives and mental health [11,12,13], we think that monitoring psychological health and intervening to prevent costly consequences or improve well-being with tailored psychological interventions are essential.

Future studies are needed to approach this active surveillance approach as a useful and concrete instrument for institutions and health policy.

Moreover, as our study contributes to the growing field of infodemiology in the pandemic context, further research could expand our research questions, analyzing and controlling for other factors that could influence word use in tweets in this pandemic period, such as geotagging, days, and seasonal weather [44,45], as well as age, gender, working status, and other sociodemographic and spatial-temporal characteristics.

### Conclusions

An increasing amount of literature has demonstrated the vast effects this pandemic is having on mental health, emotions, and cognition of the global and Italian populations. However, to the best of our knowledge, this is the first study analyzing psycholinguistic summary variables and their relationships with official COVID-19 Italian data and phases of managing the pandemic in a sample of Italian tweets during the first phases of the pandemic.

Our results show a powerful picture of the effects of COVID-19–related data and phases on emotions, analytical thinking, and somatosensory processes of Italian Twitter users: Specifically, when there was an increase in daily deaths and daily new cases, negative emotions and somatosensory words, often linked to traumatic events and PTSD symptoms, increased too. Moreover, emotional tone and analytic thinking were lower in the first phase of the pandemic, which was characterized by uncertainty, and increased during the first and second phases. As new instruments are implemented to monitor patients’ psychological status [46], having information on how the pandemic may affect the use of words with its relationships with psychosocial variables could be useful for institutions and health policies to develop specific interventions in order to mitigate the effects of this or future situations on the population’s mental health. Even if more studies are necessary, our results showed the feasibility and importance of infodemiological indicators and psycholinguistic analysis to monitor mental health–related variables in these unprecedented situations.

## Appendix

Multimedia Appendix 1 Final dataset.

Multimedia Appendix 2 Jamovi script.

## Abbreviations

LIWC: Linguistic Inquiry and Word Count
PTSD: posttraumatic stress disorder
