# Supplementary material for: Emotional Tone, Analytical Thinking, and Somatosensory Processes of a Sample of Italian Tweets During the First Phases of the COVID-19 Pandemic: Observational Study
Source: J Med Internet Res. 2021 Oct 27;23(10):e29820. doi: 10.2196/29820 (PMC8552964; doi:10.2196/29820)
Supplement: Multimedia Appendix 2 [file jmir_v23i10e29820_app2.docx]

**Syntax for emotional tone model**

gamlj::gamljGLM(

formula = toneOK ~ Phase + new_discharges + new_cases + new_deaths + new_cases:new_deaths,

data = data,

postHoc = ~ Phase,

eDesc = TRUE,

simpleVariable = new_cases,

simpleModerator = new_deaths)

**Syntax for analytical thinking model**

gamlj::gamljGLM(

formula = analyticOK ~ Phase + new_discharges + new_cases + new_deaths,

data = data,

postHoc = ~ Phase,

eDesc = TRUE)

**Syntax for somatosensory processes model**

gamlj::gamljGLM(

formula = perceptualOK ~ Phase + new_discharges + new_cases + new_deaths + new_cases:new_deaths,

data = data,

postHoc = ~ Phase,

eDesc = TRUE,

simpleVariable = new_cases,

simpleModerator = new_deaths)
